# “When you live in good health with your husband, then your children are in good health ….” A qualitative exploration of how households make healthcare decisions in Maradi and Zinder Regions, Niger

**DOI:** 10.1186/s12889-022-13683-y

**Published:** 2022-07-15

**Authors:** Sara Chace Dwyer, Sanyukta Mathur, Karen Kirk, Chaibou Dadi, Leanne Dougherty

**Affiliations:** 1 Population Council, Washington, D.C USA; 2 Conception Etudes Suivi Evaluation Appuis Formation, Niamey, Niger

**Keywords:** Child health, Family planning, Nutrition, Gender, Niger, Partner communication, Behavior change

## Abstract

**Background:**

Gender dynamics influence household-level decision-making about health behaviors and subsequent outcomes. Health and development programs in Niger are addressing gender norms through social and behavior change (SBC) approaches, yet not enough is known about how health care decisions are made and if gender-sensitive programs influence the decision-making process.

**Methods:**

We qualitatively explored how households make decisions about family planning, child health, and nutrition in the Maradi and Zinder regions, Niger, within the context of a multi-sectoral integrated SBC program. We conducted 40 in-depth interviews with married women (*n* = 20) and men (*n* = 20) between 18 and 61 years of age.

**Results:**

Male heads of household were central in health decisions, yet women were also involved and expressed the ability to discuss health issues with their husbands. Participants described three health decision-making pathways: (1^st^ pathway) wife informs husband of health issue and husband solely decides on the solution; (2^nd^ pathway) wife informs husband of health issue, proposes the solution, husband decides; and (3^rd^ pathway) wife identifies the health issue and both spouses discuss and jointly identify a solution. Additionally, the role of spouses, family members, and others varied depending on the health topic: family planning was generally discussed between spouses, whereas couples sought advice from others to address common childhood illnesses. Many participants expressed feelings of shame when asked about child malnutrition. Participants said that they discussed health more frequently with their spouses’ following participation in health activities, and some men who participated in husbands’ schools (a group-based social and behavior change approach) reported that this activity influenced their approach to and involvement with household responsibilities. However, it is unclear if program activities influenced health care decision-making or women’s autonomy.

**Conclusions:**

Women are involved to varying degrees in health decision-making. Program activities that focus on improving communication among spouses should be sustained to enhance women role in health decision-making. Male engagement strategies that emphasize spousal communication, provide health information, discuss household labor may enhance couple communication in Niger. Adapting the outreach strategies and messages by healthcare topic, such as couples counseling for family planning versus community-based nutrition messaging, are warranted.

**Supplementary Information:**

The online version contains supplementary material available at 10.1186/s12889-022-13683-y.

## Background

In Niger, under-five mortality rates are high (84 per 1,000) [1] and 44% of children are stunted due to persistent malnutrition [2]. Niger has the highest fertility rate in the world with 7.6 births per woman [2]. Yet, only 20% of married women of reproductive age are currently using a modern contraceptive method (e.g., condoms, oral contraceptives, injectables, implants, intrauterine device, tubal ligation or vasectomy) [3]. In addition to supply challenges relating to access and availability of contraceptive products [4], low unmet family planning (FP) need (16%) coupled with low contraceptive use suggests weak demand for contraception among married women [2]. Pro-natalist cultural norms, coupled with a lack of decision-making power by women for health and fertility matters, further challenge efforts to promote FP in the region [5]. Culturally defined values and norms around women’s lack of agency and resource access often limit positive health behaviors such as care seeking for sick children, decisions for healthy feeding practices, and contraceptive use [6]. According to the 2012 Niger Demographic and Health Survey, 76% of women state the husband alone should make decisions related to a woman’s health [2].

Decision-making patterns, control of household resources, and gender roles can positively affect health behaviors and outcomes [7–9]. In Niger, husbands’ encouragement for mothers to seek care for malnourished children is important in the timely decision to seek care [10] and women’s empowerment is associated with children’s dietary diversity [11]. In addition, previous studies have found protective effects on child health outcomes when a woman is able to make decisions alone or jointly with her partner [12]. Evidence suggests that care seeking for sick children follows gender norms relative to parental roles. For instance, research in West Africa has shown that mothers, given their close contact with children, are typically the first to identify illness symptoms and are primarily responsible for caregiving while fathers are more likely to determine where care is sought and to be responsible for financial costs associated with care seeking for sick children [13–16]. Women are more likely to use contraceptives following communication with their partner [17–19]. In Mali, for instance, the odds of contraceptive use were elevated among women whose partner approved of FP, who had a recent discussion with their partner and who were exposed to FP messages [20]. In Niger specifically, studies have examined the influence of gender norms and gender inequality on early child and forced marriage [21] and FP use [22, 23]. In the Maradi region of Niger, women were more likely to use modern contraception if they had discussed contraceptive use with their husband [23]. Recent social and behavior change (SBC) programs have engaged both men and women in their programming to address these inequities.

The USAID’s Resilience in the Sahel Enhanced (RISE) II program (2018–2023) builds on an earlier iteration (the RISE I program, 2012–2017) to improve priority behaviors and health outcomes in maternal, newborn, and child health, FP, nutrition, and water, sanitation, and hygiene in the Maradi and Zinder regions of Niger by targeting chronically vulnerable populations. Due to the complexity of the interactions between the underlying determinants and the gender and social norms of the RISE priority health outcomes, RISE II partners are implementing integrated SBC development programming in conjunction with humanitarian assistance. Integrated SBC approaches allow individuals to receive and discuss cross-cutting information on health and issues such as gender equality, especially as it relates to a variety of health outcomes [24, 25]. RISE II partners are using a variety of SBC approaches including community engagement, interpersonal communication (IPC) through peer group activities, and radio. IPC activities such as care groups (for women) and husbands’ schools (for men) aim to influence nutrition for pregnant and lactating women, infant young child nutrition practices, uptake of contraception, child health and water, sanitation and hygiene related behaviors [26] as well as addressing male engagement and couple’s communication. The husbands’ schools specifically bring together married men through peer group activities with health workers and cultural and religious leaders to discuss the importance of male involvement in household responsibilities, and improved couples’ communication and joint decision-making related to maternal and child health. These activities also serve as a “hub” for interrelated community wide activities including agricultural assistance, savings and loan groups, youth theater, and grandparent clubs.

This study qualitatively explores household decision-making, especially spousal communication related to child health, nutrition, and FP behaviors and how community members perceieve the gender specific RISE II SBC programming in the Maradi and Zinder regions of Niger. We use Colvin et al.'s [8] conceptual framework for care seeking of child illness which outlines household pathways to care and decision-making and refer to Kabeer’s framework [27] for women’s empowerment to guide our qualitative exploration. Previous studies have assessed women’s empowerment, including agency and access to resources, and health outcomes in low- and middle-income countries [28–30]. However, there is limited evidence from Niger and most studies focused on a single health area and did not consider how SBC programs could synergistically address gender dimensions of child health and nutrition alongside maternal and reproductive health interventions [30]. This study contributes detailed information on partner communication and household decision-making related to healthcare for multiple behaviors and describes perceptions of and reactions to initial RISE II programming related to health decision-making. This study aims to support the adaptation of integrated SBC program strategies through implementation research [31] with a particular emphasis on gender-sensitive approaches.

## Methods

### Study design and sample

We conducted a formative qualitative study to explore gender sensitive SBC approaches to address partner communication and household decision-making related to healthcare for multiple behaviors in the context of the RISE II program. The study took place in the Maradi and Zinder regions of Niger—where the RISE II program has been supporting interventions to improve health outcomes since March 2020. Maradi and Zinder are densely populated, agriculturally based regions in southern Niger compared to the northern part of the country which is largely desert and nomadic. To achieve saturation, the study team conducted 40 in-depth interviews with married men (ages 18–59) and married women (ages 18–49) with at least one child, from both monogamous and polygamous households, stratified by family size (less than 4 versus 4 or more children) [32]. Participants were not married to each other. The stratified sampling based on family size and sex aimed to capture gender dynamics and household decision-making within different family structures [33, 34]. Within each stratum, the study team randomly selected study participants from a roster of RISE II intervention participants provided by the RISE II implementing partners and contacted village health committee leaders in advance to schedule the interview. Of the 51 program participants who were contacted in advance, 40 were interviewed. The primary reason participants contacted were not interviewed was because they were absent at the time of the call.

The study team developed, piloted, and made subsequent revisions to the in-depth interview guides for the purposes of this study in collaboration with the in-country research partner Conception Etudes Suivi Evaluation Appuis Formation (CESAF) in Zinder (Supplemental file 1: Interview Guides). The interview guides included questions on health-based discussions and the health decision-making process related to experienced issues and hypothetical scenarios related to child health, child malnutrition, and FP. The guides also included questions about the specific RISE II activities with a specific emphasis on aspects of the program that addressed gender [35–37].

### Procedures

Data collection took place from December 2020 through February 2021. Male interviewers interviewed men, and female interviewers interviewed women because Islamic cultural practices require limited interaction between sexes if individuals are not related. Interviewers trained in qualitative methods described the objectives of the research, obtained written informed consent from all participants (or verbal consent if the participant was unable to write), and then administered the in-depth interview guide in Hausa to the study participants. Interviewers were fluent in both French and Hausa. A notetaker was present for each interview. Study participants were not compensated for their participation. Interviews took place in-person at the participant’s home in an outdoor and private location, and participants were provided with a mask and asked to maintain a one-meter distance throughout the interview to mitigate potential transmission of COVID-19. Interviews lasted approximately 30 min and notetakers took notes throughout the process. Interviews were recorded using a digital audio recorder and interviewers translated and transcribed the recordings into French following the discussion. CESAF reviewed a subset of audio recordings to ensure the accuracy of the translated transcripts. A total of 10 women and 10 men were interviewed in each region for a total of 40 study participants. Interviews were first conducted in Zinder and the guides were improved based on preliminary analysis before the next round of interviews were done in Maradi.

### Analysis

The study team used open coding on a sub-set of transcripts and classified emerging themes related to: [1] partner communication; [2] family communication; [3] communication outside the household; [4] gender norms; [5] health intentions; [6] access to and experience with healthcare services; and [7] exposure and perceived relevance of RISE II programming [38]. Two French-speaking staff from the study team applied codes using NVivo 12. Questions around translations from Hausa to French were confirmed with native Hausa speakers at CESAF. Prior to applying the codes across all transcripts, a subset of transcripts was coded by staff to ensure inter-rater reliability and agreement in coding across the themes. Coding agreement was 80% or above across thematic codes and the Cohen's kappa coefficient was estimated to be moderate [39]. Inconsistencies in how codes were applied were resolved after discussion with the broader study team. The team then compared findings across health areas (child health, nutrition, and FP), type of participant (male/female), and region to highlight common results across themes, specifically variation in spousal communication in health care decision-making, how individuals and couples engage family or non-family members based on the healthcare topic, and the influence of external factors, including gender dynamics, resource availability and SBC programming on health communication and gender roles within the household. The consolidated criteria for reporting qualitative research (COREQ) checklist were applied to ensure complete and transparent reporting (Supplemental file 2: COREQ checklist).

The Ministry of Public Health National Ethics Committee for Health Research in Niger provided approval for the study and consent forms (No. 017/2020/CNERS). The study also received approval from the Population Council Institutional Review Board in the United States (Protocol number 934). All methods were carried out in accordance with relevant guidelines and regulations outlined in the study protocol.

## Results

The 20 women interviewed were between 18 and 40 years of age (average age 28.6 years). Approximately three-fifths were in monogamous marriages and the average number of living children was 4.7. The 20 men interviewed were between 25 and 61 years of age (average age 42.8 years). Approximately three-fifths were in monogamous marriages and the average number of living children was 7.2. Although our sampling aimed to capture gender dynamics and household decision-making within different family structures and in different study sites, there were no major differences in our results or themes by these socio-demographic characteristics. We present overarching themes related to spousal communication, pathways to healthcare decisions, and perceived influence of SBC programs.

### Health decision-making pathways between spouses

The concept of unity or a unified decision was an important and consistent theme across male and female respondents. Most male and female participants described that they, and their spouses, arrived at the same healthcare decision. Some participants noted that while spouses could have, and voice, different opinions, they generally came to the same decision through discussion. Some women noted that it was important for spouses to come to the final decision because they were “one” and “in this together.” Men noted that when wives supported husband’s decisions, it was because wives knew that husbands tried to do the best for their families and husbands wouldn’t “suggest something that will harm the household.” Both men and women noted that if they each came to a different decision for a health issue that would create “problems in the marriage.”

To arrive at this decision/course of action for a health issue, men and women in both regions noted one of three decision-making pathways with their spouses. In the first pathway, the wife’s primary role was to provide her husband with information and the husband solely decided what to do, found the necessary financial or material resources (if needed), and identified relatives or friends who could offer support. For example, a Zinder Man described a conversation with his wife if faced with a case of child malnutrition.

*“The decision that I will take, I will order [my wife] to bring the girl to a health center, she [the mother] will take her on her back and bring her to the health center, where they provide care to the malnourished.” Zinder Man*

In the second pathway, the wife not only informed her husband about the health issue but also proposed the solution. Her husband then gave his approval and provided financial, transport, and/or emotional support identified by his wife:

*“Surely, she told me that we will have to make arrangements to solve the problem of [child’s] stomachache.” Maradi Man*

*“… [It is] the man's obligation is to go and get food for his family. At the moment, he has gone to the market, and he is thinking about us.” Maradi woman*

In the third pathway, the wife identified the health issue and then both spouses discussed how to jointly identify a solution. Below are two examples from Maradi related to couple’s decision-making for a child illness.

*“… The most recent example is that of our child's diarrhea. Since we communicate with each other and understand each other, we acted quickly to bring the child to the health center before the illness became more complicated.” Maradi Man*

*“The first thing we will do is to sit down and discuss what we should do.” Maradi Man*

Most participants acknowledged the wife as the primary person who identified child health issues because the children were “always at her side.”

*“It is me, of course, because the child is always by my side. For example, when he comes back from the market, I can explain the health situation of the child.” Maradi Woman*

While almost all men and women described one of the three pathways for decisions around personal health, child health, or malnutrition, most described the second or third pathway when it came to FP. Most said that women take a larger role in the decision to use FP because they “suffered most” from pregnancy, childbirth, and child rearing. Only a small minority suggested that it was the husband who decided whether his wife would use FP.

*“This is why women usually brings up the topic of family planning with their men first, so that they can get some rest.” Maradi Man*

*“I'll tell him: ‘I'm going to the health center about birth spacing. I want to rest. After the rest, we can continue to have children.’” Maradi Woman, 45 11 204*

Across all three pathways, the level of involvement of the wife in the decision-making process varied by household. The husband as head of household, however, played a central role in the decision-making process by either making decisions independently (pathway one), approving their spouses’ proposed solution (pathway two), or making decisions with their spouse (pathway three). A few respondents noted, at healthcare providers at times required spousal consent before the provision of contraceptives.

### Couples may discuss health decisions with others, depending on the topic

While everyone said they discussed health and healthcare with their spouses, they also involved other family members or individuals outside of the household in healthcare decision-making, depending on the topic. Many said they would talk to family members, neighbors, or close friends about common child health issues (and to some extent when asked about a hypothetical case of child malnutrition) for advice or support. Many also said they would tell their family and friends about a child illness so that they were “aware” and could learn from their own situation. For example:

*“We inform the mother-in-law so that she takes care of the rest of the children and takes care of the animals when we last there.” Maradi Woman*

*“We will also sensitize others by explaining to them that keeping a child at home is useless. The fact that we do not bring him to the health centers can cause many health problems.” Zinder Man*

Overwhelmingly however, when presented with a scenario of child malnutrition, many men and women expressed that they would be ashamed. These individuals would therefore only talk to parents, co-spouses, or close neighbors/friends who could offer advice and support to improve their child’s health. Some further explained that they would not want many people to know because they would be viewed as negligent or unable to care for their children.

*“These are people you don't have to inform, but now with the awareness we are doing, there may be others who don't want us to know that their children are malnourished just so they don't have to be asked to bring them to the health center. And now we are at a stage where people are ashamed that we know their children are malnourished because they will be seen as negligent mothers.” Maradi Woman,*

Few noted, however, that food security was a growing problem in their communities contributing to persistent malnutrition.

*“It's climate change. Sometimes just after the harvest, sometimes just one, two, three or four months later, people go into a period of food uncertainty, it's a problem.” Zinder Man*

Contraceptive use was generally only discussed between spouses compared to discussions around child illness. Few women said they may also discuss FP with a close friend and few men said they may seek advice or approval from their in-laws, especially if the in-laws were supporting the family with food, money, or childcare.

*“It is a family secret… The husband is the only one to be informed.” Maradi Woman*

*“My girlfriend with whom we sit, a close friend with whom we all say, we give each other advice, I can tell her.” Zinder Woman*

### SBC and health programming appear to influence health discussions and FP norms

Participants were exposed to the RISE II activities to varying degrees based on what was being implemented at the time of the interview and their own level of involvement. Most participants did not distinguish between activities but referred to the project as a whole. When asked whether they knew of specific activities, most reported that they were aware of the food assistance provided by RISE II, as well as the women’s care groups, and husbands schools. When asked whether the RISE II activities had any influence on couple communication and health decision-making, many men and women said that they spoke more frequently with their spouses about health since the project started. Some described how they (or their spouses) would come home after participating in a RISE II activity and share what they learned.

*“The chance we have is that our women go to the health centers a lot with this innovation brought by the HAMZARI project and it is very easy for us to initiate a discussion on health issues.” Maradi Man*

*“Now we help each other and without taboo, I told you from above that I bring water to my wife, I sweep the house if I find her very busy, I look for firewood and I even often take care of the children even if she is there.” Maradi Man*

For men in Maradi where the husband school activity was already established, many described how the husband school had a positive influence on their involvement in household tasks and in facilitating dialogue with their spouses. Men also described husband schools as a source for information and comradery.

*“Health issues are discussed at almost every moment between my wife and me, especially when we are trained on a new topic at the husband school. The most recent example is that of a delivery that she made at home... And I was very unhappy about this [home delivery] because my friends from the husband school had asked me why.” Maradi Man*

*“We always consult each other since we were sensitized through the husbands’ school, and we continue ourselves to sensitize others." Maradi Man*

Most men and women said that it was common for couples to discuss FP and echoed some of the health messaging from RISE I and RISE II activities. For instance, they noted the potential health benefits of FP for mother and children. Some participants described how breastfeeding was important for a child’s nutrition and having children too quickly would “steal the milk from one child for the next.” Many also described FP as a way for women to “rest” in-between births.

*“Many people now have understood, they make contraceptive injections.” Zinder Man*

They did not view the RISE II activities as having an influence on what decisions were made or women’s autonomy in decision-making. Both men and women reported the need to be calm, level-headed, and tactful when discussing health topics with their spouses. In a resource constrained environment such as Niger, external factors such as persistent drought can influence income and resources available to households. Some women underlined these constraints by describing how families must be patient in acting on health decisions as they/their spouses find the needed financial or material resources.

## Discussion

This paper qualitatively explores gender dynamics, specifically spousal communication and household decision-making related to reproductive and child health behaviors. This analysis adds to the growing body of literature examining the role of gender dynamics on FP and child health in the Sahel region and in Niger specifically, where the qualitative exploration of these intersections remains limited. Results from this study confirmed the role of gender dynamics in health communication and decision-making but highlighted a more nuanced view of women’s contributions to healthcare discussions. We find household communication, health decision-making, and evidence of women’s agency (and its limits) vary by household and by health topic. These insights could help tailor SBC programming based on health topic and offer potential avenues to enhance gender considerations. Additionally, this paper presents initial reflections of participant engagement with gender-integrated SBC programming applied by RISE, specifically the perceived influence of male engagement strategies, to advance couple communication and health outcomes.

A range of individual, social, and structural factors, including gender dynamics and roles influence care-seeking behaviors, especially for child health [9]. Prior literature from the Sahel region has noted the high gender inequality and poor empowerment of women [40]. Lack of women’s decision-making power within the household has been noted as a challenge for FP promotion efforts in the region [5, 20]. Confirming prior literature, male and female participants in our study reiterated women’s role in the home and in caretaking [16] and men’s role as providers and as the head of the household [15]. These gender roles and dynamics manifested in women’s role in health communication within the household. For example, in all three pathways described by participants, most highlight that women typically identify health concerns or issues because of their role in caretaking. Both men and women noted that they discuss health issues with each other and highlight women’s role in decision-making about FP and child healthcare. These results highlight women’s agency or ability to initiate care or conversations about the appropriate course of action, suggesting that women play some role in healthcare decision-making. Women’s agency, however, varied by pathway. Of the three decision-making pathways presented, in the first pathway women had limited agency and their role was to inform and bring up health-related concerns to their spouses. In the second and third pathways, women had more say. In the second pathway women were able to identify the issue and offer the solution and in the third pathway women seemed to have a role in joint decision-making. For FP in particular, respondents noted how women’s voices and choices were important, and that women took an active role in deciding whether and when to use FP. Men were central to all three decision-making pathways described by participants, especially when external resources were required to address a health problem. This is consistent with previous literature and the gender roles observed in the region [9, 10, 13].

Our study sheds light on how different SBC strategies may need to be emphasized to address reproductive health versus child health and nutrition outcomes. Most respondents in our study noted that decisions around FP are private and only discussed with close family members and generally after they have been discussed between spouses. While the importance of mothers-in-law or other family elders in fertility-related decisions has been noted in the region [41], men and women in our study emphasized that discussions around FP use were initiated by women if they were interested and ready to use them (referring to the second or third decision-making pathway). Common justifications for FP use were women’s well-being/allowing women to “rest” and the health/development of children being improved through adequate spacing. Thus, reaching couples for FP counseling could be an option in the region. While home-based couples counseling approaches can be resource intensive, other interventions that provide men and women with FP information and build skills for effective couple communication and negotiation could be useful in the region. Additionally, a more positive framing of FP, one that is oriented to help couples achieve their family goals and women’s well-being, rather than pregnancy avoidance, may be beneficial in the region. Well-designed evaluations will be needed to assess program effects, strategies, and costs of couple communication interventions in the region.

Community-level interventions may be needed around nutrition to destigmatize the issue of child malnutrition. Food insecurity (lack of food and poor food diversity) is a persistent issue in our study communities [42] and influences health and nutrition outcomes for all. Yet, men and women expressed a sense of personal shame if their child was diagnosed as malnourished. They noted being hesitant to disclose if their child was malnourished; choosing to seek out only close friends and family members who could offer advice, not ridicule. Community-level SBC interventions that destigmatize malnutrition by sharing strategies with all parents to ensure child health and nutrition in food-scarce settings may be warranted. These interventions also need to layer onto structural interventions that directly address food insecurity and increase women’s access and control over agricultural assets [43]. Additionally, given men and women’s roles in decision-making about child healthcare, interventions focused on child health and nutrition outcomes in this region may also need to consider gender-sensitive approaches [30]. Education about nutritious foods for child health, for instance, are best targeted not just for women, but men as well.

Respondents in our study noted an increased awareness of health issues and discussions with partners because of their participation in RISE II activities. Most men in Maradi described the husbands’ schools as having influenced their engagement in the household in addition to discussing health topics with their spouses. This group-based approach of male engagement seems promising to better increase men’s knowledge and awareness on health-related issues and to increase spousal communication. Our data suggests that program activities that encourage constructive spousal communication [44]— more frequent and open communication, with empathy and support for each other, to discuss possible solutions and compromises—may influence relationship quality and potentially the decision-making pathways for couples in this context. We were unable to discern, however, the influence of RISE II SBC activities on women’s autonomy and agency. For instance, female participants in our study did not explicitly mention the influence of RISE II interventions (like the women’s care groups) on their agency and empowerment. It is also unclear to what extent the project is currently synchronizing interventions by including both men and women [45].

Further implementation research is warranted to assess the design and fidelity of the gender focused programming within an integrated SBC development and humanitarian program, to explore the intent and theorized pathways of change related to gender roles, household decision-making, and health practices, and their effects on gender transformative change. Additional examination is needed to better understand how the gender-focused interventions are influencing reproductive health and child health outcomes in the RISE II program sites and for similar programs, and what are the positive or negative unintended consequences of the gender-focused SBC programming.

### Limitations

To narrow the scope of the study, we specifically assessed household decision-making as it relates to health outcomes and did not explore broader dimensions of gender empowerment such as economic and political factors which also contribute to health outcomes. While participants in our study did note that the SBC interventions under RISE II influenced greater couple communication, we are unable to fully assess if this communication and engagement in SBC programming empowered women and expanded their autonomy or freedom to make life decisions and enhanced their agency or ability to achieve their health goals. Gender norms and decision determinants may vary by ethnic group in Niger. However, our study did not seek to assess potential differences in gender norms by ethnicity. Finally, while previous studies acknowledge that household and community members beyond the couple influence health decisions [41, 46], our study did not interview the extended family (i.e., mothers-in-law, grandmothers, brothers, etc.) or friends.

## Conclusion

Previous research has shown gender dynamics are a key determinant of FP, child health, and nutrition outcomes in Niger. Like in most settings, families make health decisions based on a variety of cultural and social factors but there is no one decision-making pathway that fits all households. We find that women are involved in discussions with their husbands around health and healthcare but the degree to which this involvement translates into agency to make decisions is varied and often curtailed when resources are required to address a health problem. Different SBC strategies may be needed depending on the healthcare topic, such as couples counseling for FP versus community-based nutrition messaging. Husbands’ schools as a male engagement strategy also appear to be promising in the Niger setting, however these need to be implemented without diminishing women’s agency in the household. Additional examination is needed to ensure that gender-focused SBC programming is achieving the intended outcomes.

## Supplementary Information

**Additional file 1: Appendix B.4.** In-depth interview guide

**Additional file 2.** COREQ (Consolidated criteria for REporting Qualitative research) Checklist

## Data Availability

The datasets used and/or analyzed during the current study available from the corresponding author on reasonable request.

## Abbreviations

CESAF: Conception Etudes Suivi Evaluation Appuis Formation
FP: Family planning
IPC: Interpersonal communication
RISE: Resilience in the Sahel Enhanced
SBC: Social and behavior change
USAID: U.S. Agency for International Development
