# Supplementary material for: “When you live in good health with your husband, then your children are in good health ….” A qualitative exploration of how households make healthcare decisions in Maradi and Zinder Regions, Niger
Source: BMC Public Health. 2022 Jul 15;22:1350. doi: 10.1186/s12889-022-13683-y (PMC9283840; doi:10.1186/s12889-022-13683-y)
Supplement: Supplementary file 1 — Additional file 1: Appendix B.4. In-depth interview guide [file 12889_2022_13683_MOESM1_ESM.docx]

**Appendix B.4: In-depth maintenance guide**

**Household health care decision-making**

Thank you very much for taking the time to speak with me today. The purpose of our discussion is to learn more about how you and your family make health-related decisions. It **will be a matter of discussing not only the decisions, but also the division of responsibilities within the family.** Your answers will help us understand how the community can best meet your health needs. In our discussion today, I will present two scenarios that involve decisions about health and family. We are interested in what you and your family would do in each of these situations. We will also ask you about your experiences of participating in the activities organized by HAMZARI. We are interested in all your ideas, comments and suggestions - there are no right or wrong answers. Any information you give us will be confidential.

**A. Introduction to the discussion on the PCRD -**  **BRISE-GLACE**

***[Make sure all these questions are asked]***

For starters, I'd like to know more about you.

1. Tell me a little bit about yourself and your family?

1. ***Follow-up:***  Does your partner live with you or does he live elsewhere?
2. ***Follow-up:***  How many children do you have and can you tell me a little bit about them?
3. ***Follow-up:***  Who else lives with you?
4. Yourhusband has How many women? Are you the first, the second...?
5. [**MEN'S ONLY**] In total, with how many wives or wives do you live as a groom?
6. Tell me about the roles and responsibilities you have in your household. What types of activities do you do in the house every day?

**B. General questions about health research behaviours:**

**[Interviewer]**  Great, thank you for sharing this with me. I would like to ask you a few questions to learn more about your personal experience with the disease.

1. Think about the last time your child was sick. What symptoms have you seen?
2. When you realized he was sick, who did you talk to in your home about his illness and why?
3. How did you start the conversation?
4. What did you discuss?
5. What did this person suggest?
   1. ***Follow-up:*** What did you think of his suggestion?
6. What kind of support did this person offer you? By support, I mean financial support, emotional support(e.g. prayers). If so, say anoia little more.
   1. ***Follow-up:***  Did this person offer to help you in other ways, such as by monitoring your children or taking you to the health centre? If so, say anoia little more.
7. Were there people you didn't want to talk to about this disease, why or why not?
8. Please describe to us what you did to make him feel better?

***If the interviewee says they are going to the health centre:***

1. Tell me what you would do to get to the health centre. For example, are there things you need to do before you go? The people you need to talk to? Any arrangements to make?
   1. ***Follow-up:*** Have you had difficulty getting to the health centre? If so, explain.
   2. Ask: The difficulties of obtaining financial resources for travel?  The difficulty related to the distance to travel? access to a vehicle or motorbike? the difficulty of helping to monitor other children? If so, explain.
2. Once you have reached the health center, writeto me how did you find the quality of the services provided?
   1. Ask: What was the waiting time for services?
   2. Ask: How were you treated by the health worker?
   3. Ask: How did you findthe price of the services
3. Have you experienced difficulties in accessing medicines?

**C. Healthcenarios**

Thank you for sharing this with me. I will now read you two scenarios that involve decisions about health and family. I will address each scenario and then ask a series of questions. I would like to know more about what you and your family would do in this kind of situation. Remember that there are no right or wrong answers and think about what you would do if you were faced with this type of scenario today.

**Scenario 1: TheOmplmentary** **CDiet**

**A team of health workers went to your village to vaccinate children. After weighing your child, they inform you that his weight is less than**  **the average of his age. They**  **continue to question his**  **diet.**

***[Make sure all these questions are asked]***

**Questions and questions**

1. When you realized that the child was underweight, who did you talk to in your home about this problem and why?
2. Howwill you get the conversation going?
3. Whatare you going to talk about?
4. How do you think that person would react? What do you thinkhe or she would suggest?
5. What kind of support do you think this person would offer you? By support, I mean financial support, emotional support(e.g. prayers). If so, say anoia little more.
   1. ***Follow-up:*** Would this person offer to help you in other ways, such as by watching your children or taking you to the centre? If so, say anoia little more.
6. After talking with [enter the person's name here], are there other people **outside your home** [e.g. a friend or religious leader] that you would consult and why? If not, why not tell someone outside **your home?**
7. How will you start the conversation?
8. What are you going to talk about?
9. How do you think that person would react? What do you think shewouldsuggest?
10. What kind of support do you think this person would offer you? By support, I mean financial support, emotional support (e.g. prayers). If so, say anoia little more.
    1. ***Follow-up:***  Would this person offer to help you in other ways, such as by monitoring your children or taking you to the health centre? If so, say anoia little more.
11. Were there people you didn't want to talk to about this problem,why or why not?

**Scenario 2: Space or prevent future pregnancy**

**You [or your wife] have just given birth to a child. You [or your wife] and your child are all healthy after birth. With 4 children**   **under the age of 8, you're not sure you want more children in the future,**  **or** you think **you don't want another child right away.**

***Examples of questions and questions***

1. Is there anyone in your home you could talk to about it, and why?
   1. Follow-up: *If the participant does not want to discuss it with anyone, ask them why they do not want to discuss it with others.*
2. Howwill you get the conversation going?
3. Whatare you going to talk about?
4. How do you think that person would react? What do you think shewouldsuggest?
5. What kind of support do you think this person would offer you? By support, I mean financial support, emotional support (e.g. prayers). If so, say anoia little more.
   1. ***Follow-up: Would this person give*** you advice on what you should do? If so, say anoia little more.
6. After talking with [enter the person's name here], are there other people  **outside your home** [e.g. a friend or religious leader] that you would consult and why? If not, why not tell someone **outside**  **your home?**
7. How will you start the conversation?
8. What are you going to talk about?
9. How do you think that person would react? What do you think shewouldsuggest?
10. What kind of support do you think this person would offer you? By support, I mean financial support, emotional support (e.g. prayers). If so, say anoia little more.
    1. ***Follow-up: Would this person give*** you advice on what you should do? If so, say anoia little more.
11. Were there people you didn't want to talk to about this problem,why or why not?
12. When would you [or your wife] talk about using a family planning method?
13. Who would address this topic first and what--what could he say?
14. What would be your reaction during this conversation?
15. How would you decide [and/or your wife] whether or not to talk to a health worker?
16. What other factors might influence the decision to talk to a health worker about family planning?
17. Would this situation have been very different if you had not had children? What for?
18. Follow-up: What if you had more than 4 children? I don't know why.

**D. Reactions to DFSA activities**

**READ:** As you may know, HAMZARI provides members of the community, including women and their husbands, with information on how to improve the health of mothers and children. The project allows information to be shared through community group activities such as discussions on conference boards, videos and home visits by community health workers.

1. Can you describe all the activities you've been involved in?
2. *Follow-up:* What names have been given to this activity?
3. *Follow-up:* Who else participated in the activities (is it an individual activity, a group activity?)
4. [Ask: what about your partner? Was he involved]
5. What did you learn?
   1. [Sonde: Breastfeeding; Family Planning; Roles of Husbands and Women]
6. Thank you for giving me this information. Of the various activities carried out by the project, which were the most useful to you and why?
7. What got you interested in these activities?
8. What did your friends or other community members say about these activities?
9. What did they have to discuss and what did they think of this information?
10. What have they learned new or different?
11. Can you explain how the activity has influenced the way you **communicate with your partner** and how he/she communicates with you?
12. How have health conversations evolved between you and your partner from the time you participated in this activity to the present day?
    - 1. [*Ask:* Do you make decisions differently? Can you give an example]
13. How did you discuss health issues with your partner? Can you describe a recent example where you discussed these issues?
14. Give a few reasons why this activity has not influenced the way you communicate with your partner and how he/she communicates with you
    - 1. [Sonde: wasn't he involved in an activity, influence of other family members, influence of community members]
15. When you discuss a health topic with your partner, does your partner support your views? Why not?
    1. What are your suggestions on how the [name of the activity] could help you improve health care communication with your partner?
16. Is there an activity that has had an impact on your **roles and responsibilities** between you and your partner in the home? If so, doyou want to explain how the activity has impacted your roles and responsibilities? What about your spouse's?
17. [Ask: How have roles and responsibilities changed since you participated in this activity until today? ]
18. ***Follow-up:*** Do you describe a recent example where you both discussed it?
19. If not, are there some of thereasons why this activity did not influence the roles and responsibilities of the household between you and your spouse?
20. [Sonde: wasn't he involved in an activity, influence of other family members, influence of community members]
21. ***Follow-up:*** What are your suggestions on how the [name of the activity] could help you improve communication with your partner about roles and responsibilities within the household?

**[FEMALE RESPONDANTS ONLY]**

1. Have you participated in activities that encouraged women to participate in income-generating activities?
2. Yes
3. Not
4. If so, what types of activities? What are they talking about?
5. What did you learn?
6. What is the impact of this activity on your livelihood? Or why didn't she?
7. If you have access to your own money, how would it affect your ability to make decisions about your health care or your child's?
8. Do you have any suggestions on how to improve these activities?
